# Improper sitting posture mediates the association between single-shoulder backpack carrying and back pain in adolescents: a cross-sectional and longitudinal analysis

**DOI:** 10.3389/fpubh.2026.1857805

**Published:** 2026-06-23

**Authors:** Hong Ding, Laiguo Han, Meichen Wu, Hebao Wen, Wei Liang, Hongyu Wang

**Affiliations:** 1 College of Humanities and Health, Bengbu Medical University, Bengbu, China; 2 College of Nursing, Bengbu Medical University, Bengbu, China

**Keywords:** longitudinal observation, mediation analysis, musculoskeletal health, postural behavior, schoolbag carriage

## Abstract

**Background:**

The association between single-shoulder backpack carrying and back pain in adolescents remains unclear. This study investigated whether the degree of improper sitting posture mediates the relationship between single-shoulder backpack carrying and back pain in both cross-sectional and longitudinal analyses.

**Methods:**

This study collected data from a school-based sample covering nine provinces in China from 2021 to 2025. Back pain and backpack carrying mode were assessed through self-reporting. Improper sitting posture was scored using a three-item scale endorsed by the Chinese Ministry of Education. A cross-sectional analysis was conducted on 3,420 participants, and a longitudinal analysis was conducted on 2,423 participants. Multivariate logistic regression and Cox proportional hazards models were used to evaluate the associations between single-shoulder backpack carrying, improper sitting posture, and back pain. Mediation analysis was used to assess the mediating role of improper sitting posture in the association between single-shoulder backpack carriage and back pain. Subgroup analyses were conducted to evaluate effect modification across prespecified subgroups. Statistical significance was set at *p* < 0.05 (two-sided).

**Results:**

In the cross-sectional analysis, single-shoulder backpack carrying (OR = 1.402, 95%CI: 1.128–1.742, *p* = 0.002) and improper sitting posture (OR = 2.029, 95%CI: 1.695–2.430, *p* < 0.001) were both positively associated with back pain. Improper sitting posture accounted for 37.42% of the association between the single-shoulder backpack carrying and back pain. In the longitudinal analysis, single-shoulder backpack carrying (HR = 2.147, 95% CI: 1.565–2.945, *p* < 0.001) and improper sitting posture (HR = 1.885, 95% CI: 1.473–2.412, *p* < 0.001) were both positively associated with back pain. Improper sitting posture accounted for 11.45% of the association of single-shoulder backpack carrying with back pain.

**Conclusion:**

Single-shoulder backpack and improper sitting posture were significantly and positively associated with back pain, and improper sitting posture partially mediated the association between single-shoulder backpack carrying and back pain. These results suggest that modifying backpack carrying mode and improving improper sitting posture may be associated with a lower likelihood of back pain in adolescents.

## Introduction

Back pain is a common musculoskeletal condition characterized by discomfort or pain localized between the lower rib margin and the gluteal folds (1). It has become a significant global health problem. Studies indicate that back pain is highly prevalent among young populations, with estimates ranging from 30 to 40% in children and adolescents, and approximately three to four out of ten adolescents experiencing back pain at any given time (2). Data from the Global Burden of Disease Study further demonstrate that in 2021 alone, there were over 19 million prevalent cases of low back pain among individuals aged 10–19 years worldwide, highlighting its substantial global burden (3). In China, the burden of back pain is similarly considerable, with tens of millions of individuals affected and a continuous increase in prevalence over recent decades, making it one of the leading contributors to years lived with disability (YLDs) in the population (1). Importantly, adolescent back pain is associated with reduced physical function, decreased quality of life, and increased risk of persistent or recurrent pain later in life, imposing both individual and societal burdens (4). Beyond these immediate consequences, adolescent back pain has been shown to predict chronic pain and work related disability in adulthood (5), with evidence linking it to increased sickness absence (6), reduced labor force participation (7), and lower lifetime earnings (8). Thus, back pain during adolescence not only affects current well-being but also imposes a substantial economic burden on future society. Owing to the lack of effective curative interventions, early detection of individuals at elevated risk has drawn growing interest. Modifying known risk variables and adopting multifaceted lifestyle modifications early may help delay or reduce the likelihood of back pain.

Backpack carrying modes among adolescents typically include bilateral load carriage using both shoulder straps (double-shoulder backpack carrying) and unilateral load carriage using a single strap (single-shoulder backpack carrying). The method of carrying schoolbags also appears to influence the prevalence of back pain. A study examining the impact of single-shoulder backpack carrying found a positive association with low back pain, indicating that asymmetrical loading may contribute to discomfort and pain (9). This finding is corroborated by research, which identified double-shoulder backpack carrying as a protective factor against musculoskeletal pain, emphasizing the importance of balanced weight distribution (10). Unilateral load carriage has been shown to alter spinal alignment, increase lateral trunk deviation, and induce compensatory postural adjustments, potentially leading to musculoskeletal strain and discomfort (11). Therefore, investigating the relationship between single-shoulder backpack carrying and back pain in adolescents is essential for identifying modifiable risk factors and informing preventive strategies.

Improper sitting posture is generally defined as a sustained non-neutral spinal alignment, such as forward head posture, rounded shoulders, or asymmetrical trunk positioning, which may increase mechanical stress on the musculoskeletal system (12). In adolescents, habitual behaviors that induce asymmetrical loading are considered important contributors to such postural deviations. Single-shoulder backpack carrying introduces unilateral loading to the body, which can lead to compensatory trunk lean, pelvic obliquity, and shoulder asymmetry in order to maintain balance. These compensatory adaptations are not limited to standing or walking but may persist during seated activities, reinforcing asymmetric spinal alignment and promoting the development of poor sitting posture over time (13, 14). Studies have shown that unilateral load carriage significantly alters upper body posture and spinal alignment, increasing lateral deviation and muscular imbalance, which may predispose adolescents to adopting maladaptive postural habits (11, 15). Given that both backpack carrying mode and improper sitting posture a substantial proportion of adolescents’ daily life, understanding how single-shoulder backpack contributes to improper sitting posture is of considerable importance for clarifying potential behavioral mechanisms and informing targeted interventions.

Improper sitting posture has been consistently identified as an important factor associated with back pain in adolescents. Sustained non-neutral spinal alignment, such as forward head posture, increased thoracic kyphosis, and asymmetric trunk positioning, can lead to abnormal mechanical loading on the spine and surrounding soft tissues. These improper postures increase compressive forces on intervertebral disks, alter muscle activation patterns, and contribute to muscle fatigue and ligament strain, thereby elevating the risk of pain and discomfort (16, 17). In addition, prolonged sitting in improper sitting posture may reduce postural variability and impair neuromuscular control, further exacerbating cumulative spinal stress over time, thus predisposing to back pain (18). Adolescents may be particularly vulnerable to these effects due to ongoing musculoskeletal development and the high proportion of time spent in seated activities during school hours. Given the modifiable nature of sitting posture, examining its role in the development of back pain is of considerable importance for understanding underlying mechanisms and informing effective prevention strategies. Therefore, given the established links between asymmetric load carriage, postural alterations, and spinal stress, improper sitting posture may mediate the relationship between backpack carrying mode and back pain in adolescents.

This study examined the associations between school-related behaviors and musculoskeletal health among adolescents across nine provinces in China, with baseline data collected in 2021 and follow-up assessments completed in 2025. We tested three *a priori* hypotheses: (H1) single-shoulder backpack carrying is positively associated with back pain; (H2) single-shoulder backpack carrying is associated with improper sitting posture; and (H3) improper sitting posture mediates the association between single-shoulder backpack carrying and back pain. Findings elucidate behavioral pathways underlying adolescent back pain and support the development of targeted, school-based interventions.

## Methods

### Study design and population

**Figure 1:**
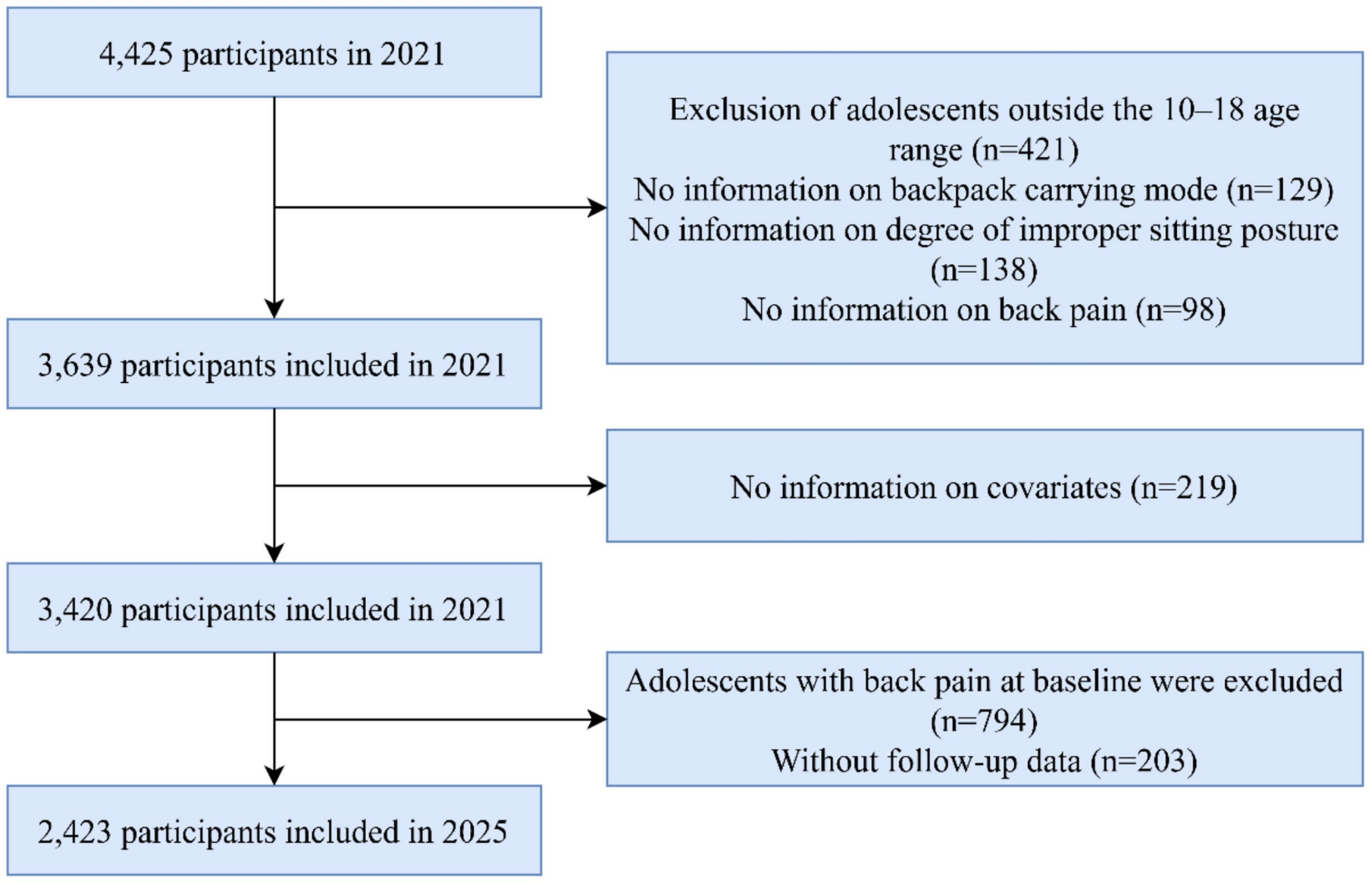
Flowchart of the participants selection process.

The study was conducted in nine provinces in China in 2021. A total of 4,425 adolescents were surveyed. Figure 1 shows the selection process of the study population. At baseline (Wave 1, 2021), 4,425 participants were assessed for the baseline analysis. Participants were excluded based on the following criteria to ensure methodological accuracy: children outside the 10–19 age range (*n* = 421), incomplete backpack carrying mode (*n* = 129), missing degree of improper sitting posture (*n* = 138), missing data of back pain (*n* = 98). A total of 3,639 participants remained. Incomplete covariate information was excluded (*n* = 219), and a total of 3,420 individuals were retained in cross-sectional study. For the longitudinal analysis, we excluded children with back pain (*n* = 794). During follow-up waves (from 2021 to 2025), 203 participants were excluded due to loss to follow-up during the period. Thus, 2,423 participants were included.

### Assessments

#### Back pain

Back pain was self-reported in 2021 using a single-item question: “Do you often experience back pain?” Responses were coded dichotomously (yes/no).

#### Backpack carrying mode

The backpack carrying modes of teenagers are divided into double-shoulder backpack carrying and single-shoulder backpack carrying. These data were collected in 2021.

#### Degree of improper sitting posture

The degree of improper sitting posture among adolescents was defined using three evidence-informed criteria from China’s Ministry of Education for proper reading and writing posture: (1) upright seated position, with eye-to-book distance of 33–35 cm (≈1 foot), chest-to-desk distance of ~1 fist, and finger-to-pen-tip distance of ~3 cm (≈1 inch); (2) appropriate pen-holding angle—40°–50° for pencils or pens, and near-vertical for brush pens; and (3) avoidance of reading while head-tilted, supine, walking, or in moving vehicles or vessels. To quantify the degree of improper sitting posture, participants self-reported whether they usually violated each of the three criteria. For each criterion violated, a score of 1 was assigned. The total score was then summed, yielding a range from 0 (no violations, indicating proper sitting posture) to 3 (violation of all three criteria, indicating the most improper sitting posture). This total score was used as a continuous variable in all analyses, with higher scores representing a greater degree of improper sitting posture.

#### Control variables

The baseline questionnaire collected demographic and health data, including age and sex (male or female). Age was recorded and grouped for analysis. Anthropometric measurements, specifically height and weight, were collected using standardized protocols to calculate body mass index (BMI). Participants were classified into four BMI categories according to World Health Organization (WHO) criteria: underweight (BMI < 18.5 kg/m^2^), normal weight (BMI 18.5–24.9 kg/m^2^), overweight (BMI 25.0–29.9 kg/m^2^), and obese (BMI ≥ 30.0 kg/m^2^). Daily sleep duration was also assessed and categorized into three groups: <7 h, 7–9 h, and >9 h.

### Statistical analysis

Statistical analyses were conducted in R (v4.3.3). Descriptive statistics summarized participant characteristics: continuous variables as mean ± SD; categorical variables as frequency (percentage). Spearman rank correlations assessed bivariate associations among key variables. Multivariable Cox and logistic regression models evaluated the associations of single-shoulder backpack carrying and improper sitting posture with back pain, yielding hazard ratios (HRs) and odds ratios (ORs), respectively. The proportional hazards assumption for the Cox models was tested using Schoenfeld residual analysis. The global test and variable-specific tests were performed, and a non-significant *p*-value (*p* > 0.05) indicates that the assumption is not violated. Two models were fitted: an adjusted model (sex, age, BMI, daily sleep duration) and an unadjusted model. Subgroup analyses explored effect heterogeneity across demographic, behavioral, and health-related strata. Owing to the number of statistical tests we performed, a Bonferroni correction was used for multiple testing. Missing covariate data affected 219 participants (6.0% of the cross-sectional sample). We used complete-case analysis without imputation, as missingness was low and met the MCAR assumption—confirmed by nonsignificant differences (all *p* > 0.05) in age, sex, BMI, sleep duration, single-shoulder backpack carrying, and back pain status between those with complete data and those excluded (Supplementary Table S1). Sensitivity analysis was omitted, per standard practice under MCAR with minimal missingness. Mediation analysis was performed to examine whether improper sitting posture mediated the association between single-shoulder backpack carrying and back pain. The analysis followed the product-of-coefficients approach based on regression models. Three separate regression models were fitted to estimate: (1) the total association of single-shoulder backpack carrying with back pain, (2) the association of single-shoulder backpack carrying with the mediator (improper sitting posture), and (3) the association of the mediator with back pain, with all models adjusted for covariates. The indirect (mediation) association was calculated as the product of the coefficients from the second and third models, and the proportion mediated was derived as the ratio of the indirect association to the total association. Bootstrap resampling with 5,000 replicates was used to obtain bias-corrected 95% confidence intervals for the indirect association; mediation was considered present if the interval excluded zero. Separate mediation analyses were conducted for the cross-sectional data (using logistic regression for back pain outcomes) and for the longitudinal data (using Cox regression for time-to-event outcomes). In the longitudinal analysis, the mediator and covariates were measured at baseline, and back pain was assessed at follow-up.

## Results

### Characteristics of the study participants at baseline

A total of 3,420 participants were included (Table 1), with 794 (23.2%) reporting back pain. Compared with the non-back pain group (*n* = 2,626), the back pain group (*n* = 794) exhibited significant differences across multiple baseline characteristics. In terms of demographic characteristics, a higher proportion of females were observed in the back pain group (24.8% vs. 21.8% in the non-back pain group, *p* = 0.041). Age distribution also differed significantly, with the back pain group having a greater proportion of older adolescents: 25.4% were aged 13–15 years and 33.3% were aged 16–18 years, compared to 37.4 and 25.4% in the non-back pain group, respectively (*p* < 0.001). Regarding anthropometric measures, the back pain group showed a higher prevalence of obesity (26.3% vs. 23.9% in the non-back pain group) and a lower prevalence of underweight (22.0% vs. 28.0%), with a significant overall difference in BMI distribution (*p* = 0.004). Lifestyle factors revealed notable disparities: shorter daily sleep duration was more common in the back pain group (34.8% slept <7 h vs. 25.2% in the non-back pain group), while longer sleep (>9 h) was less frequent (16.0% vs. 20.5%, *p* < 0.001). Additionally, single-shoulder backpack carrying was more prevalent in the back pain group (32.2% vs. 21.7%, *p* < 0.001). Finally, the back pain group demonstrated a significantly higher degree of improper sitting posture (2.05 ± 0.45 vs. 1.88 ± 0.48, *p* < 0.001).

**Table 1:** Baseline characteristics of selected participants (*N* = 3,420).

Variables	Total	Non-back pain	Back pain	*p*-value
Total	3,420	2,626	794	
Sex, *n* (%)				0.041
Male	1,780 (52.0)	1,392 (78.2)	388 (21.8)	
Female	1,640 (48.0)	1,234 (75.2)	406 (24.8)	
Age, *n* (%)				<0.001
10 ~ 12 years old	1,073 (31.4)	909 (84.7)	164 (15.3)	
13 ~ 15 years old	1,921 (56.2)	1,433 (74.6)	488 (25.4)	
16 ~ 18 years old	426 (12.5)	284 (66.7)	142 (33.3)	
BMI, *n* (%)				0.004
Underweight	649 (19.0)	506 (78.0)	143 (22.0)	
Normal weight	1,687 (49.3)	1,265 (75.0)	422 (25.0)	
Overweight	749 (21.9)	608 (81.2)	141 (18.8)	
Obese	335 (9.8)	247 (73.7)	88 (26.3)	
Daily sleep duration, *n* (%)				<0.001
<7 h	908 (26.5)	592 (65.2)	316 (34.8)	
7–9 h	2,256 (66.0)	1,819 (80.6)	437 (19.4)	
>9 h	256 (7.5)	215 (84.0)	41 (16.0)	
Backpack carrying mode, *n* (%)				<0.001
Double-shoulder backpack carrying	2,917 (85.3)	2,285 (78.3)	632 (21.7)	
Single-shoulder backpack carrying	503 (14.7)	341 (67.8)	162 (32.2)	
Degree of improper sitting posture	1.92 ± 0.48	1.88 ± 0.48	2.05 ± 0.45	<0.001

### The association between backpack carrying mode and degree of improper sitting posture with back pain

Table 2 presents the results of the cross-sectional regression analysis examining the associations of backpack carrying mode and degree of improper sitting posture with back pain. In the unadjusted model, single-shoulder backpack carrying was significantly associated with back pain compared with double-shoulder backpack carrying (OR = 1.718, 95% CI: 1.397–2.112, *p* < 0.001). Similarly, the degree of improper sitting posture was positively associated with back pain (OR = 2.186, 95% CI: 1.834–2.607, *p* < 0.001). After adjusting for sex, age, BMI, and daily sleep duration, the associations remained statistically significant. The adjusted OR for single-shoulder backpack carrying was 1.402 (95% CI: 1.128–1.742, *p* = 0.002) relative to double-shoulder carrying. Furthermore, the degree of improper sitting posture remained significantly associated with back pain (Adjusted OR = 2.029, 95% CI: 1.695–2.430, *p* < 0.001).

**Table 2:** Regression analysis of backpack carrying mode and degree of improper sitting posture on back pain in cross-sectional analysis.

Variables	No.	Unadjusted		Adjusted	
		OR (95% CI)	*p*	OR (95% CI)	*p*
Backpack carrying mode	3,420				
Double-shoulder backpack carrying	2,917	1 (Ref)		1 (Ref)	
Single-shoulder backpack carrying	503	1.718 (1.397, 2.112)	<0.001	1.402 (1.128, 1.742)	0.002
Degree of improper sitting posture	3,420	2.186 (1.834, 2.607)	<0.001	2.029 (1.695, 2.430)	<0.001

After adjusting for sex, age, BMI and daily sleep duration.

Table 3 presents the results of the longitudinal regression analysis regarding the associations between backpack carrying mode, improper sitting posture, and back pain. In the unadjusted model, single-shoulder backpack carrying was significantly associated with back pain compared with the double-shoulder backpack carrying reference group (HR = 1.904, 95% CI: 1.422–2.556, *p* < 0.001). Similarly, the degree of improper sitting posture showed a significant positive association with back pain (HR = 1.763, 95% CI: 1.397–2.226, *p* < 0.001). After adjusting for sex, age, BMI, and daily sleep duration, the associations remained statistically significant. The adjusted HR for single-shoulder backpack carrying was 2.147 (95% CI: 1.565–2.945, *p* < 0.001) relative to double-shoulder carrying. Furthermore, the degree of improper sitting posture remained significantly associated with back pain (adjusted HR = 1.885, 95% CI: 1.473–2.412, *p* < 0.001). The proportional hazards assumption was satisfied (global test *p* = 0.599; all individual variables *p* > 0.05), as shown in Supplementary Table S2.

**Table 3:** Regression analysis of backpack carrying mode and degree of improper sitting posture on back pain in longitudinal analysis.

Variables	No.	Unadjusted		Adjusted	
		HR (95% CI)	*p*	HR (95% CI)	*p*
Backpack carrying mode	2,423				
Double-shoulder backpack carrying	2,119	1 (Ref)		1 (Ref)	
Single-shoulder backpack carrying	304	1.904 (1.422, 2.556)	<0.001	2.147 (1.565, 2.945)	<0.001
Degree of improper sitting posture	2,423	1.763 (1.397, 2.226)	<0.001	1.885 (1.473, 2.412)	<0.001

After adjusting for sex, age, BMI and daily sleep duration.

Supplementary Tables S3 and S6 present subgroup analyses of single-shoulder backpack carrying and back pain. In the cross-sectional study (Supplementary Table S3), age significantly modified this association (interaction *p* = 0.005). In the longitudinal study (Supplementary Table S6), no significant effect modification was observed across any subgroup (all interaction *p* > 0.0125).

Supplementary Tables S4 and S7 present subgroup analyses of single-shoulder backpack carrying and degree of improper sitting posture. Both the cross-sectional and longitudinal studies showed consistent associations across all subgroups, with no significant interactions (all *p* > 0.0125).

Supplementary Tables S5 and S8 present subgroup analyses of degree of improper sitting posture and back pain. In the cross-sectional study (Supplementary Table S5), no significant effect modification was detected (all interaction *p* > 0.0125). In the longitudinal study (Supplementary Table S8), BMI significantly modified the association (interaction *p* = 0.006).

### Degree of improper sitting posture mediated the association between backpack carrying mode and back pain

Table 4 presents the association analysis of backpack carrying mode and degree of improper sitting posture with back pain in cross-sectional analysis. The single-shoulder backpack carrying was positively associated with back pain (*r* = 0.088, *p* < 0.001). The single-shoulder backpack carrying was positively associated with degree of improper sitting posture (*r* = 0.119, *p* < 0.001). The degree of improper sitting posture is positively correlated with back pain (*r* = 0.151, *p* < 0.001).

**Table 4:** Association analysis of backpack carrying mode and degree of improper sitting posture with back pain in cross-sectional analysis.

Variables	Single-shoulder backpack carrying	Degree of improper sitting posture	Back pain
Single-shoulder backpack carrying	1		
Degree of improper sitting posture	0.119^***^	1	
Back pain	0.088^***^	0.151^***^	1

^*^p < 0.05, ^**^p < 0.01, ^***^p < 0.001, and so on.

Table 5 presents findings from the longitudinal analysis. Single-shoulder backpack carrying was positively associated with back pain (*r* = 0.088, *p* < 0.001). The single-shoulder backpack carrying was positively associated with degree of improper sitting posture (*r* = 0.113, *p* < 0.001). The degree of improper sitting posture is positively correlated with back pain (*r* = 0.097, *p* < 0.001).

**Table 5:** Association of backpack carrying mode and degree of improper sitting posture with back pain in longitudinal analysis.

Variables	Single-shoulder backpack carrying	Degree of improper sitting posture	Back pain
Single-shoulder backpack carrying	1		
Degree of improper sitting posture	0.113^***^	1	
Back pain	0.088^***^	0.097^***^	1

^*^p < 0.05, ^**^p < 0.01, ^***^p < 0.001, and so on.

The association coefficients between the key variables ranged from 0.088 to 0.151, indicating weak but statistically significant associations.

**Figure 2:**
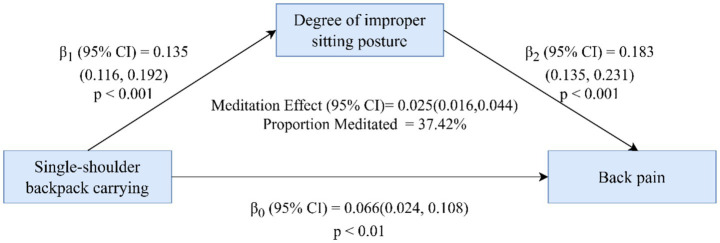
Conceptual framework of the cross-sectional mediation model. β_0_ represented the total association of single-shoulder backpack carrying on adolescents’ back pain; β_1_ represented the association of single-shoulder backpack carrying on degree of improper sitting posture; and β_2_ represented the association of degree of improper sitting posture on adolescents’ back pain. The mediation association was computed as the product β_1_ × β_2_, and the proportion mediated was calculated as (β_1_ × β_2_)/β_0_.

In cross-sectional analysis, single-shoulder backpack carrying demonstrated a statistically significant positive total association with back pain (β_0_ = 0.066, 95% Cl: 0.024, 0.108, *p* < 0.01). Degree of improper sitting posture partially mediated this relationship, with a mediation association of 0.025 (95% Cl: 0.016, 0.044), representing 37.42% of the overall association variability. The indirect pathway from single-shoulder backpack carrying to back pain through degree of improper sitting posture is depicted in Figure 2.

**Figure 3:**
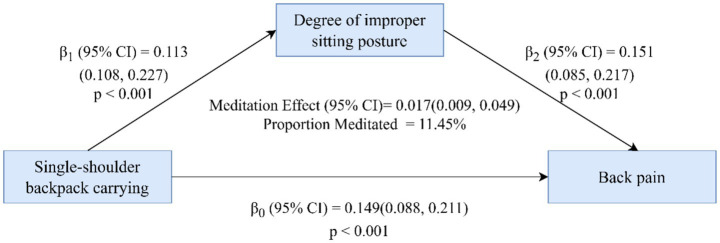
Conceptual framework of the longitudinal mediation model. β_0_ represented the total association of single-shoulder backpack carrying on adolescents’ back pain; β_1_ represented the association of single-shoulder backpack carrying on degree of improper sitting posture; and β_2_ represented the association of degree of improper sitting posture on adolescents’ back pain. The mediation association was computed as the product β_1_ × β_2_, and the proportion mediated was calculated as (β_1_ × β_2_)/β_0_.

The longitudinal bootstrap analysis also detected a significant total association of single-shoulder backpack carrying with back pain (β_0_ = 0.149, 95% Cl: 0.088, 0.211, *p* < 0.001). In this model, degree of improper sitting posture functioned as a partial mediator, exhibiting a mediating association of 0.017 (95% Cl: 0.009, 0.049), which accounted for 11.45% of the total association variance. The corresponding mediation framework is presented in Figure 3.

## Discussion

This study, based on data from Chinese adolescents from 2021 to 2025, indicates that single-shoulder backpack carrying was positively associated with back pain and improper sitting posture, while improper sitting posture was also positively associated with back pain. Improper sitting posture played a partial mediating role between single-shoulder backpack carrying and back pain. Subgroup analyses revealed that age significantly modified the association between single-shoulder backpack carrying and back pain in the cross-sectional analysis, whereas BMI significantly influenced the association between improper sitting posture and back pain in the longitudinal analysis. Thus supporting our original hypothesis.

Previous studies have shown that single-shoulder backpack carrying was associated with increased back pain in adolescents (19). In addition, asymmetric loading leads to elevated localized pressure and contributes to upper back pain development in children (20). In the present study, we confirmed previous findings and found a positive association between single-shoulder backpack carrying and back pain in adolescents through cross-sectional (OR = 1.402, 95%CI: 1.128–1.742, *p* = 0.002) and longitudinal (HR = 2.147, 95% CI: 1.565–2.945, *p* < 0.001) analyses. Unilateral load carriage has been shown to increase spinal compressive and shear forces, particularly in the lumbar region, due to uneven force transmission and trunk stabilization demands (21). This asymmetric loading requires continuous activation of contralateral trunk muscles to maintain balance, which may lead to muscle overuse, fatigue, and reduced load-sharing capacity across spinal structures (22). In addition, sustained imbalance can impair proprioceptive control and postural stability, resulting in inefficient movement patterns and increased cumulative stress on intervertebral disks and passive tissues (23). Over time, these biomechanical and neuromuscular alterations may contribute to cumulative microtrauma, degenerative changes, and increased nociceptive sensitivity, thereby increasing the likelihood of back pain (24).

Previous studies have shown that unilateral backpack loading leads to postural asymmetry in adolescents (25). In addition, asymmetric load adversely affects postural features in younger children (26). In the present study, single-shoulder backpack carrying was positively associated with improper sitting posture in adolescents. Our study focused on adolescents and further on sitting posture rather than general postural changes. Although our posture measure reflects self-reported habits rather than direct spinal alignment, a speculative behavioral pathway could be that repeated unilateral loading promotes asymmetric muscle activation patterns, which may reinforce improper sitting habits over time. However, this interpretation remains hypothetical because we did not measure muscle activity or spinal angles. The following discussion of possible mechanisms is based on previous literature, not on direct measurements from our study. Over time, such adaptations may recalibrate the sensorimotor system, including proprioceptive feedback and central postural regulation, thereby promoting the persistence of asymmetric alignment even during seated tasks (27). In addition, prolonged exposure to asymmetric loading may contribute to changes in spinal stiffness and passive tissue properties, which can further constrain neutral posture and reinforce maladaptive sitting patterns (28). These combined neuromuscular and biomechanical alterations suggest that single-shoulder backpack carrying may not only induce immediate postural deviations but also facilitate the consolidation of improper sitting habits through motor learning processes. Because our sitting posture variable does not capture true biomechanical alignment, these mechanistic suggestions require confirmation using objective posture assessment.

Previous studies have reported that prolonged slumped sitting increases trunk muscle fatigue (29). Additionally, a cross-sectional study found that greater degrees of slump in sitting posture are positively associated with higher levels of back pain (30). Moreover, improper sitting posture is a risk factor for musculoskeletal pain (31). In the present study, we confirmed previous studies, and found improper sitting posture was positively associated with back pain in adolescents through cross-sectional (OR = 2.029, 95%CI: 1.695–2.430, *p* < 0.001) and longitudinal (HR = 1.885, 95% CI: 1.473–2.412, *p* < 0.001) analyses. Furthermore, improper sitting posture may act as a mediator in the association between single-shoulder backpack carrying and back pain, with a mediation association of 0.025 (95% Cl: 0.016, 0.044), representing 37.42% of the overall association variability in the cross-sectional study. In the longitudinal analysis, the mediation association was 0.017 (95%CI: 0.009, 0.049), accounting for 11.45% of the total association variance. Given that our posture measure is a self-reported habit score, we cannot test direct biomechanical mechanisms. However, previous experimental studies have reported that prolonged sitting in flexed or asymmetrical postures has been shown to impair paraspinal muscle oxygenation and promote localized muscle fatigue, reducing the capacity of active stabilizing systems to support the spine (32). In addition, sustained static loading can compromise intervertebral disk nutrition by limiting fluid exchange, leading to decreased disk resilience and increased susceptibility to mechanical stress (33). These changes may be accompanied by low-grade inflammatory responses and peripheral sensitization, which can amplify pain perception over time. If replicated with objective posture measurement in adolescents, such mechanisms could explain the observed association between self-reported poor sitting habits and back pain.

This study has potential important public health implications for adolescent musculoskeletal health, though the weak association sizes warrant caution. Our findings suggest that modifying backpack carrying mode and sitting posture may represent a practical approach associated with a lower likelihood of back pain in adolescents. Public health efforts could consider school-based interventions that recommend the use of dual-shoulder backpacks, and promote proper sitting posture during prolonged classroom activities. These findings support the need for school-based health programs, including ergonomic education and posture training, to establish guidelines for appropriate backpack use and classroom sitting practices.

Future research should further clarify the temporal relationships among backpack carrying mode, sitting posture, and back pain using more rigorous longitudinal designs. The use of objective measurement tools, such as wearable devices or posture-monitoring systems, would improve the accuracy of exposure assessment and reduce reliance on self-reported data. Future studies should also incorporate more detailed information on backpack use, including load, carrying duration, and frequency, to better reflect the actual situation. In addition, expanding the study population to different regions and age groups would further strengthen the generalizability of the findings.

### Advantages and limitations

This study possesses several notable strengths. First, the longitudinal design with a relatively large sample size enhances the robustness of the findings and allows for better assessment of temporal relationships. Second, by integrating backpack carrying mode, sitting posture, and back pain within a unified analytical framework, this study provides a more comprehensive understanding of their interrelationships rather than examining these factors in isolation. Third, the inclusion of subgroup analyses further strengthens the study by identifying potential effect modifiers, offering more nuanced insights into population heterogeneity.

However, several limitations warrant consideration. First, the use of self-reported measures for backpack carrying mode, sitting posture, and back pain may introduce recall bias and misclassification. Second, back pain was assessed using a single binary question, which may lead to misclassification, for example by including mild or transient pain, and does not capture clinically relevant dimensions such as intensity or disability. Future research should adopt validated multidimensional pain scales. In addition, improper sitting posture was self-reported using three binary criteria, risking recall and social desirability bias. Future studies should use direct observation to minimize measurement bias. Third, reverse causality cannot be ruled out, as adolescents with back pain may be more likely to adopt single-shoulder backpack carrying or improper sitting posture. Fourth, missing data may have introduced selection bias and affect the validity of the findings. Fifth, we lacked detailed anthropometric measures of biotype, including trunk length, shoulder width, and pelvic dimensions, which may modify the biomechanical response to unilateral load carriage and sitting posture. Adjustment for BMI only partially addresses this issue. Future studies should incorporate these measurements to better understand effect modification. Sixth, this study did not collect several important potential confounders, including physical activity level, screen time, actual backpack weight, and socioeconomic status. These factors may influence both exposure and outcome; their omission may result in residual confounding, and no causal relationship can therefore be established. Seventh, the measure of “improper sitting posture” was based on three self-reported ergonomic habits, not direct biomechanical alignment. Thus, it reflects a behavioral habit score rather than true postural alignment. Without objective validation, the mediation outcomes should be interpreted as statistical associations among self-reported habits, not evidence of biomechanical pathways. Eighth, although the study used a longitudinal design—with the mediator and covariates assessed at baseline and back pain at follow-up—both backpack carrying mode and sitting posture were measured only once. These behaviors likely change over the 4-year follow-up, so repeated assessments would be needed to accurately capture temporal mediation. Finally, the study population was limited to adolescents in specific regions of China, which may affect the generalizability of the findings to other populations.

## Conclusion

This study employed both cross-sectional and longitudinal designs. Our findings demonstrated that single-shoulder backpack carrying was positively associated with improper sitting posture and back pain, while improper sitting posture was also positively associated with back pain. Notably, improper sitting posture partially mediates the association between single-shoulder backpack carrying and back pain, revealing a potential behavioral pathway linking asymmetric load carriage to musculoskeletal pain. These findings identify modifiable targets associated with a lower likelihood of back pain in adolescents, specifically reducing single-shoulder backpack use and improving sitting posture.

## Data Availability

The original contributions presented in the study are included in the article/Supplementary material, further inquiries can be directed to the corresponding author.
