# Supplementary material for: Improper sitting posture mediates the association between single-shoulder backpack carrying and back pain in adolescents: a cross-sectional and longitudinal analysis
Source: Front Public Health. 2026 Jun 23;14:1857805. doi: 10.3389/fpubh.2026.1857805 (PMC13337700; doi:10.3389/fpubh.2026.1857805)
Supplement: Supplementary file 1 [file Supplementary_file_1.DOCX]

Supplementary Table 1. Comparison of participant characteristics between those with complete covariate data and those excluded due to missing covariates

| **Variables** | **Complete data**  **(n=3,420)** | **Excluded due to missing covariates**  **(n=219)** | **P‑value** |
| --- | --- | --- | --- |
| Sex, n(%) |  |  | 0.072 |
| Male | 1,780 (52.0) | 106 (48.4) |  |
| Female | 1,640 (48.0) | 113 (51.6) |  |
| Age, n(%) |  |  | 0.086 |
| 10–12 years old | 1,073 (31.4) | 74 (33.8) |  |
| 13–15 years old | 1,921 (56.2) | 118 (53.9) |  |
| 16–18 years old | 426 (12.5) | 27 (12.3) |  |
| BMI, n(%) |  |  | 0.094 |
| Underweight | 649 (19.0) | 45 (20.5) |  |
| Normal weight | 1,687 (49.3) | 102 (46.6) |  |
| Overweight | 749 (21.9) | 48 (21.9) |  |
| Obese | 335 (9.8) | 24 (11.0) |  |
| Daily sleep duration, n(%) |  |  | 0.068 |
| ＜7 h | 908 (26.5) | 67 (30.6) |  |
| 7–9 h | 2,256 (66.0) | 139 (63.5) |  |
| ＞9 h | 256 (7.5) | 13 (5.9) |  |
| Back pain, n(%) |  |  | 0.077 |
| Yes | 794 (23.2) | 60 (27.4) |  |
| No | 2,626 (76.8) | 159 (72.6) |  |
| Backpack carrying mode, n(%) |  |  | 0.091 |
| Double‑shoulder | 2,917 (85.3) | 182 (83.1) |  |
| Single‑shoulder | 503 (14.7) | 37 (16.9) |  |

Note: No significant differences were observed for any variable (all p>0.05p>0.05), supporting the assumption that data were missing completely at random (MCAR).

Supplementary Table 2. Test of proportional hazards assumption for Cox regression models (longitudinal analysis)

| **Variable** | **χ²** | **df** | **p‑value** |
| --- | --- | --- | --- |
| Single‑shoulder backpack carrying | 0.82 | 1 | 0.365 |
| Degree of improper sitting posture | 1.24 | 1 | 0.265 |
| Sex (female vs. male) | 0.31 | 1 | 0.578 |
| Age (continuous) | 2.05 | 1 | 0.152 |
| BMI (continuous) | 0.96 | 1 | 0.327 |
| Daily sleep duration (7–9 h vs. ＜7 h) | 1.68 | 1 | 0.195 |
| Daily sleep duration (＞9 h vs. ＜7 h) | 0.44 | 1 | 0.507 |
| Global test | 5.50 | 7 | 0.599 |

Note: Schoenfeld residual test was used. A non‑significant p‑value (p > 0.05) indicates that the proportional hazards assumption is not violated.

Supplementary Table 3. Subgroup analysis of backpack carrying mode on back pain in cross-sectional analysis

| **Variables** | ***N*** | **Back pain** | ***OR (95% CI)*** | ***P for interaction*** |
| --- | --- | --- | --- | --- |
| Sex |  |  |  | 0.103 |
| Male | 1780 | 388(21.8) | 2.047 (1.512, 2.773) |  |
| Female | 1640 | 406(24.8) | 1.450 (1.092, 1.924) |  |
| Age |  |  |  | 0.005 |
| 10–12 years old | 1073 | 164(15.3) | 1.320 (0.600, 2.904) |  |
| 13–15 years old | 1921 | 488(25.4) | 1.293 (1.012, 1.652) |  |
| 16–18 years old | 426 | 142(33.3) | 3.497 (2.016, 6.064) |  |
| BMI |  |  |  | 0.397 |
| Underweight | 649 | 143(22.0) | 1.627 (1.012, 2.614) |  |
| Normal weight | 1687 | 422(25.0) | 1.726 (1.305, 2.283) |  |
| Overweight | 749 | 141(18.8) | 1.492 (0.918, 2.427) |  |
| Obese | 335 | 88(26.3) | 3.394 (1.485, 7.758) |  |
| Daily sleep duration |  |  |  | 0.922 |
| ＜7h | 908 | 316(34.8) | 1.518 (1.100, 2.096) |  |
| 7–9h | 2256 | 437(19.4) | 1.520 (1.136, 2.033) |  |
| ＞9h | 256 | 41(16.0) | 1.876 (0.696, 5.056) |  |

Supplementary Table 4. Subgroup analysis of backpack carrying mode on degree of improper sitting posture in cross-sectional analysis

| **Variables** | ***N*** | **Degree of improper sitting posture** | ***β (95% CI)*** | ***P for interaction*** |
| --- | --- | --- | --- | --- |
| Sex |  |  |  | 0.347 |
| Male | 1780 | 1.93±0.49 | 0.188 (0.119, 0.256) |  |
| Female | 1640 | 1.91±0.47 | 0.144 (0.084, 0.204) |  |
| Age |  |  |  | 0.067 |
| 10–12 years old | 1073 | 1.82±0.49 | 0.036 (-0.114, 0.185) |  |
| 13–15 years old | 1921 | 1.96±0.47 | 0.121 (0.069, 0.172) |  |
| 16–18 years old | 426 | 1.96±0.49 | 0.254 (0.126, 0.383) |  |
| BMI |  |  |  | 0.194 |
| Underweight | 649 | 1.90±0.51 | 0.199 (0.092, 0.306) |  |
| Normal weight | 1687 | 1.93±0.49 | 0.179 (0.116, 0.242) |  |
| Overweight | 749 | 1.92±0.46 | 0.129 (0.036, 0.222) |  |
| Obese | 335 | 1.90±0.44 | -0.023 (-0.202, 0.155) |  |
| Daily sleep duration |  |  |  | 0.160 |
| ＜7h | 908 | 1.96±0.51 | 0.208 (0.129, 0.287) |  |
| 7–9h | 2256 | 1.92±0.45 | 0.116 (0.059, 0.172) |  |
| ＞9h | 256 | 1.75±0.58 | 0.180 (-0.063, 0.422) |  |

Supplementary Table 5. Subgroup analysis of degree of improper sitting posture on back pain in cross-sectional analysis

| **Variables** | ***N*** | **Back pain** | ***OR (95% CI)*** | ***P for interaction*** |
| --- | --- | --- | --- | --- |
| Sex |  |  |  | 0.544 |
| Male | 1780 | 388(21.8) | 2.089 (1.642, 2.658) |  |
| Female | 1640 | 406(24.8) | 2.331 (1.798, 3.021) |  |
| Age |  |  |  | 0.110 |
| 10–12 years old | 1073 | 164(15.3) | 1.561 (1.093, 2.231) |  |
| 13–15 years old | 1921 | 488(25.4) | 2.096 (1.665, 2.638) |  |
| 16–18 years old | 426 | 142(33.3) | 2.893 (1.814, 4.614) |  |
| BMI |  |  |  | 0.776 |
| Underweight | 649 | 143(22.0) | 2.151 (1.460, 3.170) |  |
| Normal weight | 1687 | 422(25.0) | 2.205 (1.735, 2.802) |  |
| Overweight | 749 | 141(18.8) | 1.909 (1.251, 2.913) |  |
| Obese | 335 | 88(26.3) | 2.861 (1.508, 5.427) |  |
| Daily sleep duration |  |  |  | 0.131 |
| ＜7h | 908 | 316(34.8) | 2.668 (1.978, 3.598) |  |
| 7–9h | 2256 | 437(19.4) | 1.850 (1.454, 2.355) |  |
| ＞9h | 256 | 41(16.0) | 1.704 (0.948, 3.064) |  |

Supplementary Table 6. Subgroup analysis of backpack carrying mode on back pain in longitudinal analysis

| **Variables** | ***N*** | **Back pain** | ***OR (95% CI)*** | ***P for interaction*** |
| --- | --- | --- | --- | --- |
| Sex |  |  |  | 0.824 |
| Male | 1259 | 140(11.1) | 1.918 (1.185, 3.106) |  |
| Female | 1164 | 223(19.2) | 2.054 (1.431, 2.949) |  |
| Age |  |  |  | 0.091 |
| 10–12 years old | 774 | 98(12.7) | 3.127 (1.447, 6.760) |  |
| 13–15 years old | 1242 | 135(10.9) | 1.769 (1.172, 2.671) |  |
| 16–18 years old | 407 | 130(31.9) | 3.764 (2.065, 6.861) |  |
| BMI |  |  |  | 0.193 |
| Underweight | 463 | 99(21.4) | 1.201 (0.651, 2.215) |  |
| Normal weight | 1171 | 213(18.2) | 2.499 (1.710, 3.652) |  |
| Overweight | 563 | 34(6.0) | 2.825 (1.294, 6.165) |  |
| Obese | 226 | 17(7.5) | 2.189 (0.448, 10.694) |  |
| Daily sleep duration |  |  |  | 0.653 |
| ＜7h | 626 | 142(22.7) | 2.113 (1.360, 3.282) |  |
| 7–9h | 1618 | 199(12.3) | 1.733 (1.149, 2.614) |  |
| ＞9h | 179 | 22(12.3) | 2.949 (0.850, 10.240) |  |

Supplementary Table 7. Subgroup analysis of backpack carrying mode on degree of improper sitting posture in longitudinal analysis

| **Variables** | ***N*** | **Degree of improper sitting posture** | ***β (95% CI)*** | ***P for interaction*** |
| --- | --- | --- | --- | --- |
| Sex |  |  |  | 0.348 |
| Male | 1259 | 1.93±0.49 | 0.083 (-0.003, 0.170) |  |
| Female | 1164 | 1.91±0.47 | 0.137 (0.066, 0.207) |  |
| Age |  |  |  | 0.309 |
| 10–12 years old | 774 | 1.82±0.49 | 0.061 (-0.045, 0.167) |  |
| 13–15 years old | 1242 | 1.96±0.47 | 0.112 (0.027, 0.198) |  |
| 16–18 years old | 407 | 1.96±0.49 | 0.173 (0.075, 0.272) |  |
| BMI |  |  |  | 0.227 |
| Underweight | 463 | 1.90±0.51 | 0.131 (0.012, 0.250) |  |
| Normal weight | 1171 | 1.93±0.49 | 0.115 (0.041, 0.189) |  |
| Overweight | 563 | 1.92±0.46 | 0.203 (0.049, 0.357) |  |
| Obese | 226 | 1.90±0.44 | -0.106 (-0.316, 0.104) |  |
| Daily sleep duration |  |  |  | 0.062 |
| ＜7h | 626 | 1.96±0.51 | 0.199 (0.104, 0.293) |  |
| 7–9h | 1618 | 1.92±0.45 | 0.066 (-0.004, 0.135) |  |
| ＞9h | 179 | 1.75±0.58 | 0.035 (-0.220, 0.290) |  |

Supplementary Table 8. Subgroup analysis of degree of improper sitting posture on back pain in longitudinal analysis

| **Variables** | ***N*** | **Back pain** | ***OR (95% CI)*** | ***P for interaction*** |
| --- | --- | --- | --- | --- |
| Sex |  |  |  | 0.602 |
| Male | 1259 | 140(11.1) | 1.915 (1.332, 2.754) |  |
| Female | 1164 | 223(19.2) | 1.687 (1.241, 2.295) |  |
| Age |  |  |  | 0.080 |
| 10–12 years old | 774 | 98(12.7) | 1.504 (0.975, 2.322) |  |
| 13–15 years old | 1242 | 135(10.9) | 1.535 (1.061, 2.220) |  |
| 16–18 years old | 407 | 130(31.9) | 2.850 (1.758, 4.620) |  |
| BMI |  |  |  | 0.006 |
| Underweight | 463 | 99(21.4) | 1.089 (0.681, 1.741) |  |
| Normal weight | 1171 | 213(18.2) | 2.556 (1.861, 3.511) |  |
| Overweight | 563 | 34(6.0) | 1.108 (0.558, 2.198) |  |
| Obese | 226 | 17(7.5) | 0.898 (0.303, 2.666) |  |
| Daily sleep duration |  |  |  | 0.749 |
| ＜7h | 626 | 142(22.7) | 1.929 (1.298, 2.867) |  |
| 7–9h | 1618 | 199(12.3) | 1.813 (1.324, 2.484) |  |
| ＞9h | 179 | 22(12.3) | 1.361 (0.609, 3.043) |  |
